# Supplementary material for: Ruminal Bacterial Community Successions in Response to Monensin Supplementation in Goats
Source: Animals (Basel). 2022 Sep 4;12(17):2291. doi: 10.3390/ani12172291 (PMC9454474; doi:10.3390/ani12172291)
Supplement: Supplementary file 1 [file animals-12-02291-s001.zip › Table S1.docx]

**Table S1.** Ingredients and chemical composition of the experimental diet

| Item | % (DM) |
| --- | --- |
| Ingredients |  |
| Alfalfa hay | 33.3 |
| Corn silage | 26.8 |
| Soybean meal | 10.8 |
| Crushed corn | 28.0 |
| Salt | 0.4 |
| CaCO_3_ | 0.3 |
| CaHPO_4_ | 0.3 |
| Vitamin-mineral premix^1^ | 0.1 |
| Chemical composition |  |
| DM | 49.3 |
| OM | 93.8 |
| NDF | 33.9 |
| ADF | 23.9 |
| CP | 14.7 |

^1^Vitamin-mineral premix (per kg): 950 mg of Zn, 650 mg of Cu, 600 mg of Mn, 430 mg of Fe, 45 mg of I, 30 mg of Se, 20 mg of Co, 800 mg of vitamin E, 450 mg of nicotinic acid, 120,000 IU of vitamin A, and 45,000 IU of vitamin D.
