# Supplementary figures and images for: Ruminal Bacterial Community Successions in Response to Monensin Supplementation in Goats
Source: Animals (Basel). 2022 Sep 4;12(17):2291. doi: 10.3390/ani12172291 (PMC9454474; doi:10.3390/ani12172291)

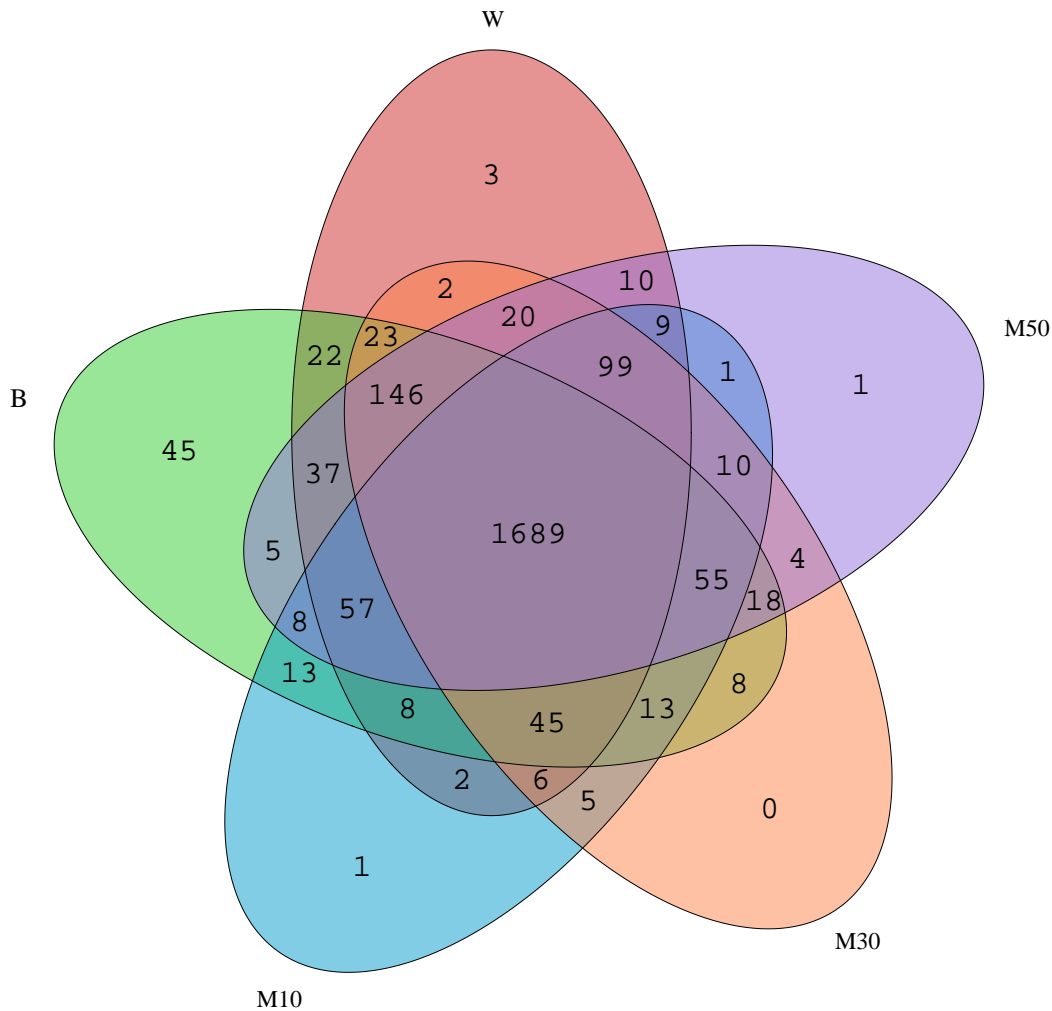

Supplement: Supplementary file 1 [file animals-12-02291-s001.zip › Figure S1.pdf]

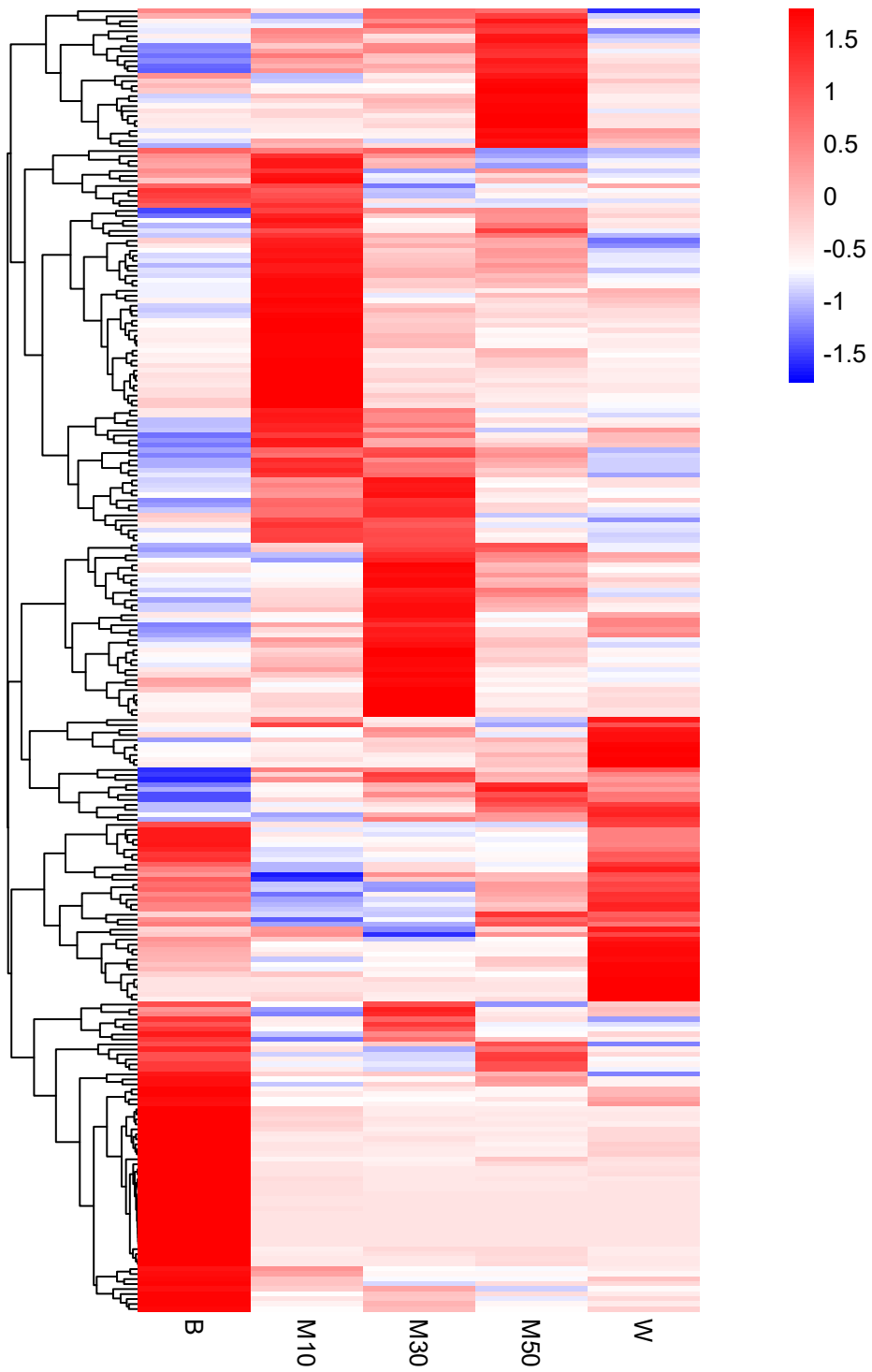

Supplement: Supplementary file 1 [file animals-12-02291-s001.zip › Figure S2.pdf]

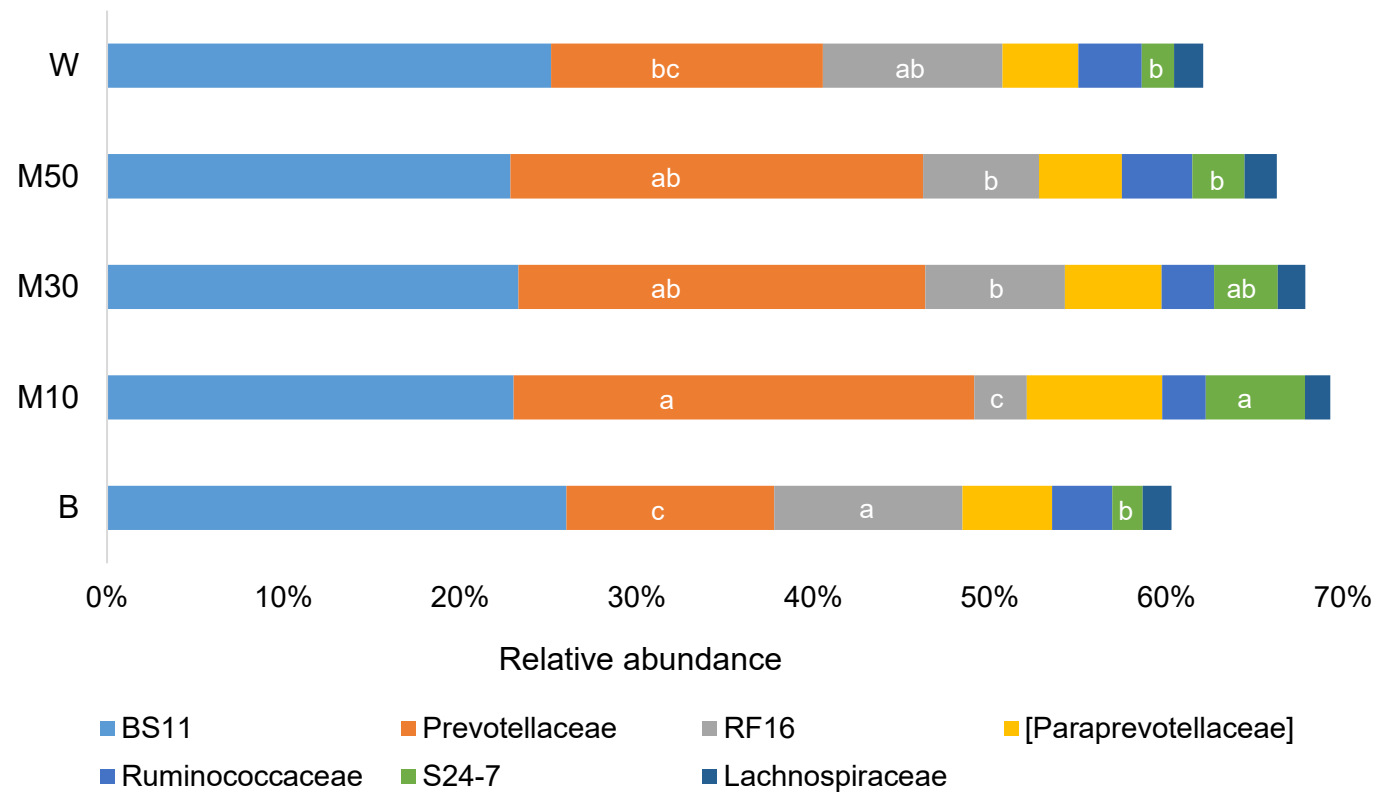

Supplement: Supplementary file 1 [file animals-12-02291-s001.zip › Figure S3.pdf]
